# Butterfly Species Richness and Diversity in the Trishna Wildlife Sanctuary in South Asia

**DOI:** 10.1673/031.013.7901

**Published:** 2013-08-10

**Authors:** Joydeb Majumder, Rahul Lodh, B. K. Agarwala

**Affiliations:** Ecology & Biodiversity Laboratories, Department of Zoology, Tripura University, Suryamaninagar, 799022, Tripura, India

**Keywords:** habitat selection, moist deciduous forest, richness estimator

## Abstract

Several wildlife sanctuaries in the world are home to the surviving populations of many endemic species. Trishna wildlife sanctuary in northeast India is protected by law, and is home to the last surviving populations of Asian bison (*Bos gorus* Smith), spectacle monkey (*Trachypithecus phayrie* Blyth), capped langur (*Trachypithecus pileatus* Blyth), slow loris (*Nycticebus coucang* Boddaert), wild cat (*Felis chaus* Schreber), and wild boars (*Sus scrofa* L.), among many other animals and plants. The sanctuary was explored for species richness and diversity of butterflies. A six-month-long study revealed the occurrence of 59 butterfly species that included 21 unique species and 9 species listed in the threatened category. The mixed moist deciduous mature forest of the sanctuary harbored greater species richness and species diversity (39 species under 31 genera) than other parts of the sanctuary, which is comprised of regenerated secondary mixed deciduous forest (37 species under 32 genera), degraded forests (32 species under 28 genera), and open grassland with patches of plantations and artificial lakes (24 species under 17 genera). The majority of these species showed a distribution range throughout the Indo-Malayan region and Australasia tropics, and eight species were distributed in the eastern parts of South Asia, including one species, *Labadea martha* (F.), which is distributed in the eastern Himalayas alone. Estimator Chao 2 provided the best-predicted value of species richness. The steep slope of the species accumulation curve suggested the occurrence of a large number of rare species, and a prolonged gentle slope suggested a higher species richness at a higher sample abundance. The species composition of vegetation-rich habitats showed high similarity in comparison to vegetation-poor habitats.

## Introduction

Recent studies of biodiversity in relation to ecosystem functioning have suggested that species diversity sometimes enhances productivity and stability of ecosystems (Naeem et al. 1994; Tilman et al. 1996). Positive relationships have been found between butterfly diversity and plant diversity (Thomas and Malorie 1985; Leps and Spitzer 1990). This relationship is particularly true in tropical regions, where insects show high abundance and species diversity (Price 1997). Due to richness in vegetation, the northeastern region of India is home to a rich diversity of butterflies, among other insects (Kunte 1997; Alfred et al. 2002), and it is also part of one of the mega biodiversity hotspots of the world (Myers et al. 2000). A review of literature suggests that 76 species of butterfly were previously recorded from the state of Tripura (10,490 sq km), northeast India (Mandai et al. 2002; Agarwala et al. 2010; Majumder et al. 2011). Among other northeastern states, 104 species of butterfly from Meghalaya (22429 sq. km), 695 species from Sikkim (7096 sq. km), and 962 species from Assam (78438 sq. km) have been recorded (Evans 1932; Talbot 1939; Wynter-Blyth 1957; Haribal 1992). Evidently, the knowledge base of lepidopteran fauna and their distribution in different habitats is uneven and still scanty from this part of India.

In this study, a detailed inventory was carried out to document the butterfly species richness and diversity of Trishna wildlife sanctuary (TWLS) in Tripura in relation to its habitat, which is comprised of mixed moist deciduous and evergreen forests with patches of long grasses. At the time of this study, 22 species were known from this sanctuary (Roy Choudhury et al. 2011) without regard to their abundance, distribution pattern, and habitat preference. Considering the fact that Trishna sanctuary is known to have 230 tree species, 110 species of shrubs, 400 species of herbs, and 150 species of climbers (www.incrediblenortheastindia.com), the assumption was that new data on spatial scale over a period of time would help to ascertain the true status of species richness and diversity in diverse habitats of the Trishna wildlife sanctuary, which is home to the Asian population of bison (*Bos gorus* Smith), and 116 species of resident and migratory birds, reptiles like cobra and king cobra snakes, and mammals like wild boar (*Sus scrofa* L.), spectacle monkey (*Trachypithecus phayrie* Blyth), capped langur (*Trachypithecus pileatus* Blyth), slow loris (*Nycticebus coucang* Boddaert) and wild cat (*Felis* chaus Schreber) (www.tripura.nic.in/trishna/intro.html). This study also intended to bring out any hitherto not recorded threatened taxa of butterfly from this natural preserve of wildlife in this part of south Asia.

## Materials and Methods

### Study area

**Figure 1.**
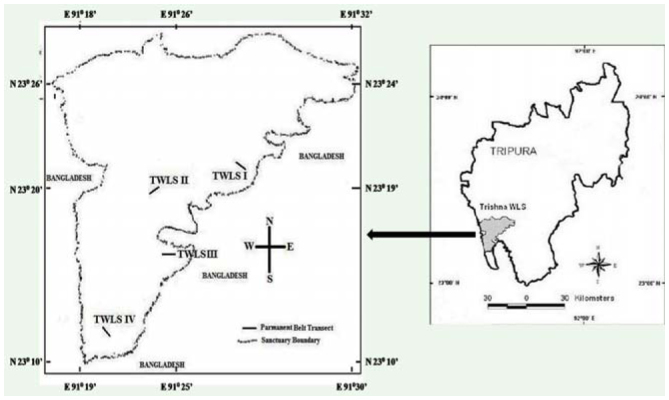
Location map of Trishna Wildlife Sanctuary. High quality figures are available online.

Trishna wild life sanctuary is situated in the south district of Tripura state between 23° 26.137' N and 91° 28.184' E and has an altitudinal gradient of 51–82 m a.s.l. (Figure 1). The total sanctuary area is 194.71 km^2^ and is delimited on the east and west sides by the international boundary with Bangladesh. The forest cover of the sanctuary consists of dense primary forest (62%) dominated by *Shorea robusta* Roth, *Dipterocarpus turbinatus* C.F.Gaertn, and *Terminalia belliraca* (Gaertn) trees, degraded forest (18%) dominated by *Toona ciliata* M. J. Roem, *Albizia procera* Durazz, a large number of shrubs, herbs and climbers, and the remaining 20% is bushy forest of bamboos, sedges, long grasses, and shrubs like *Microcos paniculata* L. of Chinese origin, *Chromolaena odorata* (L.) King and H.E. Robins of North American origin, and *Lantana camara* L. of Neotropical origin, among others. The sanctuary has a number of perennial water rivulets and patches of grasslands. The climatic condition is tropical, with a minimum rainfall of 3.58 mm in December and a maximum of 508.20 mm in July. The average minimum and maximum temperature recorded in the region is 6.8° C in January and 37.70° C in June, respectively.

This study was conducted in four distinct habitats of TWLS based on habitat type, topography, and intensity of anthropogenic pressure. A two-day scouting survey was carried out in April 2010 to identify the sampling sites, one each in four habitat types (Table 1). TWLS I (near an ecotourism site) was characterized by mature, secondary mixed moist deciduous forest dominated by *Shorea, Dipterocarpus,* and *Microcos* plants, with multilayered understory vegetation (sedges, grasses, and ferns) and a canopy height of 15 m to 20 m. The major human pressures were fodder and fuel wood collection by inhabitants settled on the periphery of the sanctuary. Two artificial lakes were present on each side of TWLS I. TWLS II (near a medicinal plant nursery) was characterized by regenerated, secondary mixed moist deciduous forest dominated by *Dipterocarpus* but few *Shorea* trees, and little understory, delimited by profuse growth of herbs and shrubs at the edges. The canopy height was between 3 m and 8 m, and the site was surrounded by paddy (*Oryza sativa* L.) fields. Wild boars inhabited this site and caused uprooting of understory vegetation. TWLS III (near a bison watch tower) was characterized by degraded forest with growths of herbs, creeper grasses, and small to large patches of several bamboo species (*Bambusa tulda* Roxbergii, *B. balcooa:* Roxbergii, and *Meloccana baccifera:* (Roxburgh) Kurtz ex Skeels) with very little understory vegetation. The forest floor was covered with leaf litter of 10 cm to 25 cm thickness. TWLS IV (a bison feeding ground) was devoid of big and old trees, and was an open type forest dominated by exotic long grasses (*Pennisetum purpureum* Schumach) and bamboos (*B. tulda, B. balcooa,* and *M. baccifera*) for grazing of bison. An artificial lake and cashewnut (*Anacardium occidentale* L.) plantation on a small scale were other features of this site.

**Table 1.**
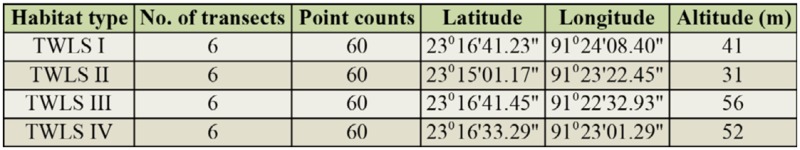
Transect information of four habitat types of Trishna Wildlife Sanctuary.

### Butterfly census

Field surveys for butterfly fauna were conducted from May 2010 to October 2010 by a modified Pollard Walk Method (Pollard 1977). One km long and 5 m wide permanent belt transects, one each in four sampling sites, representing a gradient of vegetation complex in four habitat types, were laid, and butterfly censuses were made between 08:00 and 11:00 local time on four successive days. This was repeated at 30-day intervals, maintaining the same spatial scale in the four sampling sites. Thus, butterfly censuses were performed six times in as many months at each of the four habitats of TWLS. The cumulative area of monthly survey was 20 km^2^, which represents 10.27% of the TWLS (194.71 km^2^). Point counts at 100-m-intervals were made along each transect to record butterfly species and their number. Walking pace was kept slow but at uniform speed, with a view to record the maximum richness and diversity. Stops were made along transect lines when an exact identification of the species was not possible, and the specimens were then either photographed or captured by butterfly nets for closer examination and identification. A few of the specimens that could not be identified in the field were brought to the laboratory for identification, or were referred to the subject experts. During field study, collection data comprised of dates, location, time, species, and number of individuals of each butterfly species were recorded separately for each sampling site. Vouchers of all field-collected specimens were deposited in the Ecology and Biodiversity Laboratories, Department of Zoology, at Tripura University.

**Table 2.**
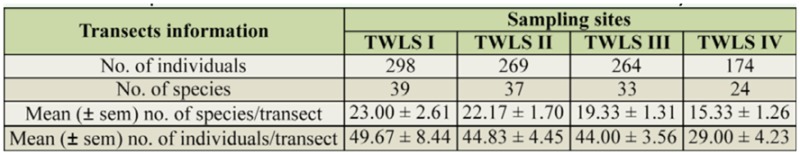
Number of individuals and species of butterflies recorded in Trishna Wildlife Sanctuary.

### Butterfly identification and geographic range

Identification, nomenclature, and geographic distributions of collected butterflies followed Evans (1932), Wynter-Blyth (1957), Arora and Mondai (1981), Tsukada (1982), Otsuka (1988), Haribal (1992), Antram (2002), and Kehimkar (2008). Classifications of butterflies in this study were based on Ackery (1984). The geographic distribution ranges of different butterfly species were categorized on a numerical scale of 1–5 (smallest to largest) with some modifications to fit within the study area: (1) Eastern Himalayas (Sikkim to Assam); (2) Northeastern India and northern Indochina (up to Northern Myanmar); (3) Indo-Malayan region (India, including Andaman Island, Myanmar, and Malaysia); (4) IndoAustralian (Australasian tropics) including all India, Myanmar, and up to Srilanka; (5) Paleotropics (most of Asia north of the Himalayan ranges apart from Africa north of the Sahara and all of Europe).

### Data analysis

Raw data from the field were used to reveal species richness (Menhinik index), species diversity (Shannon-Weiner index), component of dominance (Simpson dominance index), and relative abundance of different species in a sampling site (Pielou's evenness index) (Magurran 1988). Comparisons in species composition between different forest habitats were estimated using single linkage cluster analysis based on Bray-Curtis similarity (McAleece 1998). The species recorded in this study were ranked based on relative abundance of individual species and also according to their known geographical range of distribution. The raw data of species richness counts of six months from each sampling site were pooled to get rarefaction curves for comparison of estimated species richness between the habitats. Sampling completeness was calculated as a ratio of observed species richness to the richness estimate, and was expressed as a percentage (Sorensen et al. 2002). Biodiversity Pro version 2 (Lambshead et al. 1997) was used to determine diversity indices, cluster analysis, species accumulation curve, rarefaction curves, and species richness estimates. Rank abundance diagram and species richness index were determined by Origin version 5 (Microcal Software, Inc., www.microcal.com) and PAST version 2 (Hammer et al. 2001), respectively. The Kruskal-Wallis test was used to compare species composition between different habitat types.

**Table 3.**
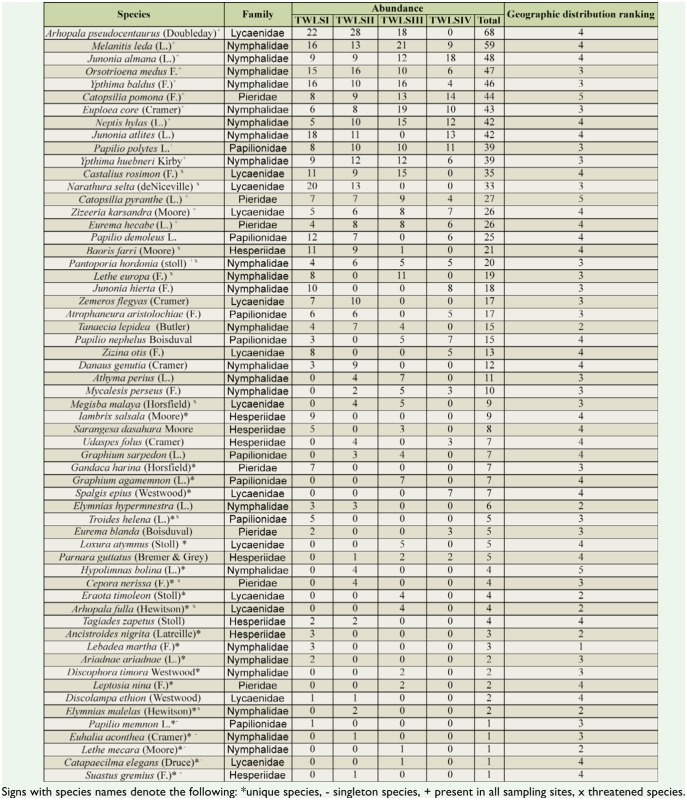
List of butterfly species, their abundance, ranking, and geographic distribution ranking recorded in Trishna Wildlife Sanctuary.

## Results

### Butterfly fauna

A total of 1005 butterflies representing 59 species belonging to 48 genera and 5 families were recorded in this study (Table 2, 3). A maximum of 39 species and 298 individuals of butterflies were recorded in TWLS-I, which was dominated by mature mixed moist deciduous forest, and minimum of 24 species and 174 individuals were recorded in TWLS-IV, which was dominated by long grasses and bamboos (Table 2). Sampling sites TWLS II and TWLS III were represented by 37 and 33 species, respectively, but without any significant difference (Kruskal-Wallis Test: *H* = 0.18, p= 0.681) between the two sites. Among the four sites, TWLS I and TWLS II showed significant differences with TWLS IV in terms of species composition (TWLSI : *H* = 6.19, *p* = 0.018; TWLS II: *H* = 4.52, *p* = 0.045). Out of 59 species, 21 species (35.59%) were recorded from one of the four sampling sites only and, hence, these were considered ‘unique’species for the purpose of this study. These included five ‘singleton’ species (Table 3). Another 14 species (23.73%) were found to be common to all the sampling sites. Nine species (15.25%) were recorded as ‘threatened’ or ‘endangered’ as per Part IV of Schedule I and Schedule II of the Indian Wildlife Protection Act 1972 (Anonymous 2006), and another one species, *Troides helena* (L.), was listed under CITES Appendix II (Collins and Morris 1985).

**Table 4.**
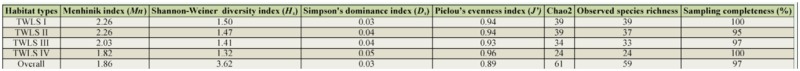
Diversity parameters and species richness estimates of butterfly communities in the four habitat types.

### Butterfly diversity

Family-wise distribution of butterflies showed that members of Nymphalidae dominated the collection (23 species and 492 individuals) followed by Lycaenidae, Papilionidae, Hesperiidae, and Pieridae, in that order (Table 3).

The diversity parameters of butterflies showed variations in the four sampling sites. In general, the four sampling sites showed high species richness and diversity of butterflies, very low dominance of species, and high evenness of distribution (Table 4). Specifically, the mature mixed moist deciduous forest at TWLS I showed maximum diversity (*H_s_*= 1.50) and least dominance (*D_s_* = 0.03) of butterflies, whereas the habitat with long grasses, bamboos, and plantation crops (TWLS IV) showed minimum diversity (*H_s_* = 1.32) and highest dominance (*D_s_* = 0.05) of butterflies. Evenness of distribution in all the study sites was found to be high (*J* = 0.93 to 0.96). Species diversity in regenerated mixed moist deciduous forest with little understory vegetation (TWLS II) and in secondary mixed moist forest with patches of bamboos (TWLS III) were lower than TWLS I but higher than TWLS IV.

### Species ranking

A ranking of 59 species according to their geographical distribution showed that 26 species were distributed in the Indo-Malayan region (rank 3), another 22 species in the Australasian tropics (rank 4), 3 species in Paleotropics (rank 5), and the remaining 8 species showed restricted geographical distribution, which included 7 species distributed in northeastern India and northern Indo-China up to northern Myanmar (rank 2), and the other species, *Labadea martha* (F.), was distributed in the eastern Himalayas (rank 1) only (Table 3).

**Figure 2.**
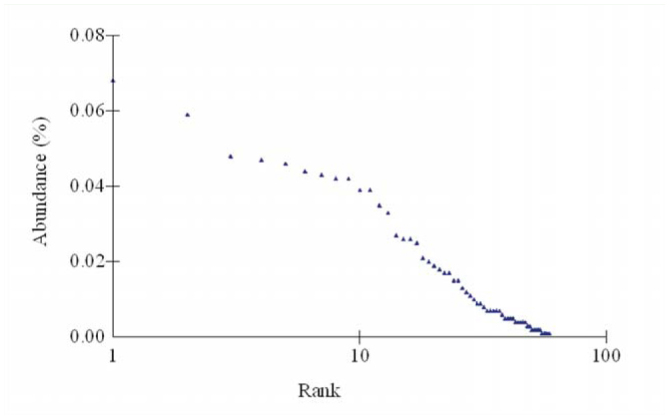
Rank abundance diagram of butterfly species recorded at Trishna Wildlife Sanctuary. High quality figures are available online.

The distribution of ranks according to the abundance of different butterfly species showed that 59 species were distributed into 33 ranks. *Amblypodia centaurus* (Doubleday) and *Melanitis leda* (L.) showed relative abundances of 6.77% and 5.87% respectively. Together, these species accounted for 127 (12.64%) of the individuals encountered in this study. The next 21 species showed abundances in the range of 1.69–4.78%, and together accounted for 67.06% of the total abundance. Another 31 butterfly species together accounted for 19.80% of total abundance. The remaining 5 species were found to be singletons (Table 3), and these contributed 0.05% of the total abundance (Figure 2).

### Richness estimates

**Figure 3.**
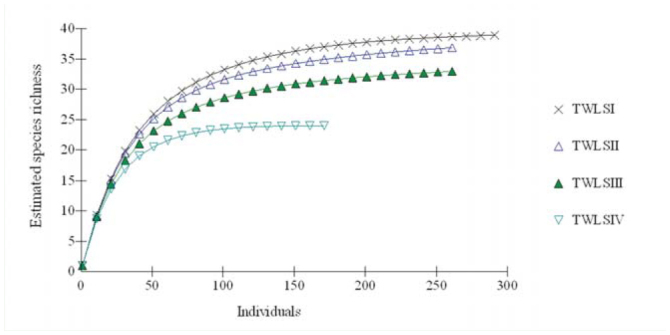
Sample based rarefaction curves of estimated species richness at four sampling sites of Trishna Wildlife Sanctuary. High quality figures are available online.

Species richness estimate, using Chao 2, was found to give the best estimate for the samples of this study. Estimation of species richness in the four habitats of TWLS by Chao 2 showed expected richnesses that were very close to the observed values (Table 4). The overall estimate of species richness from the four habitats was higher by 2 species than the observed value. This was also evident from the high values of the sampling completeness of this study, which varied in a narrow range between 95% and 100% between four habitats, and which was 97% overall. Rarefaction curves from the four habitats showed quick rises at first and than either leveled off (TWLS IV) or approached asymptote gently (TWLS I, II, and III) (Figure 3).

### Butterfly species composition

**Figure 4.**
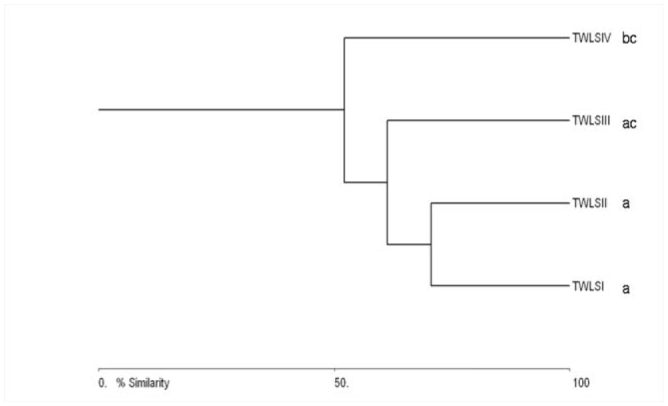
Single linkage cluster analysis between habitat types based on Bray-Curtis similarity; abbreviations denote: TWLSI = Trishna Wildlife Sanctuary I, TWLSII = Trishna Wildlife Sanctuary II, TWLSIII = Trishna Wildlife Sanctuary III, TWLSIV = Trishna Wildlife Sanctuary IV. Dissimilar letters following habitat types indicate significant differences by Kruskal-Wallis test (*p* < 0.05). High quality figures are available online.

The cluster analysis based on Bray-Curtis single linkage similarity index demonstrated the differences and similarities between the butterflies species composition recorded in four habitat types. The open habitat of long grasses and bamboos (TWLS IV) stood out clearly from the other 3 habitats and showed linkage at 50.86% similarity, which represented the lowest similarity. The degraded forest habitat (TWLS III) was linked at 58.16% similarity to the cluster of habitats of primary forest (TWLS I) and regenerated forest (TWLS II), which showed the highest similarity in species composition (70.55%) (Figure 4).

## Discussion

This is the first study on the distribution and abundance of butterflies in the quasievergreen and moist deciduous forests of TWLS. The 59 recorded species in this sanctuary compares favorably with the 71 species found in Aralam wildlife sanctuary in Kerala, south India, which has similar habitats (Sreekumar and Balakrishnan 2001). All the species recorded in Aralam wildlife sanctuary (55 sq. km) were ‘generalist’ and none of them were considered to be threatened or of rare occurrence. In contrast, eight species, i.e., *Lethe europa* (F.), *Cepora nerissa* (F.), *Castalius rosimon* (F.), *Narathura selta* (de Niceville), *Pantoporia hordonia* (stoll), *Megisba malaya* (Horsfield), *Arhopala fulla* (Hewitson), and *Baoris farri* (Moore) recorded in TWLS were enlisted in the Indian Forest Act 1972 under Schedule I & II, and another species, *Troides helena* L., was listed in Appendix -II of CITES, and these are protected by law.

Overall species abundance and richness revealed that Nymphalidae was the most speciose and individualized family, Pieridae was the least speciose family, and Hesperidae was the least individualized family in the study area. In the context of tropical environment, similar patterns of species abundance and richness were reported from the western ghats and western coast of India (Kunte 1997; Eswaran and Pramod 2005; Padhye et al. 2006; Krishnakumar et al. 2007; Raut and Pendharkar 2010), from the Silent Valley National Park, Kerala, south India (Mathew and Rahamathulla 1993), and in the Parambikulam wildlife sanctuary, south India (Sudheendrakumar et al. 2000). The dominance of Nymphalidae species may be attributed to their being polyphagous, which helps these butterflies to live in a variety of habitats, and also because many species of this family are active fliers, which helps them to forage larger areas. However, in the present study, Pieridae was poorly represented in comparison to studies made in other sanctuaries of India, which reported members of Hesperiidae to be least represented. One of the possible reasons for this difference could be due to the difficulties in observing Hesperiidae butterflies because of their dull color and ability to fly rapidly following any disturbance.

Fifty-nine species recorded in this study showed unequal distribution of abundance in the four habitats of TWLS. Several studies have reported the influence of habitat disturbance on abundance of butterflies (Hill et al. 1995). In the present study, 13 species having a geographical distribution ranking of 3–5 were present in all four habitats. Another 21 species showed low abundance and habitat specificity (Table 3). TWLS IV contained one of these, *Spalgis epius* (Westwood), whereas TWLS I, TWLS II, and TWLS III contained 7, 5, and 8 unique species, respectively. However, 6 of these were truly restricted in their geographic distribution, and the other 15 species showed wide geographical distribution. Geographically restricted species are considered habitat specific, show low ecological tolerance, prefer undisturbed to least disturbed habitats, and have high conservation value in comparison to widely distributed species, which show high ecological tolerance, occur in a gradient of vegetation complex, share food resources over a wide geographical range, and are of low conservation value (Spitzer et al. 1997). In the present study, species poor habitat TWLS IV contained one unique species, *S. epius,* which is aphidophagous. The results clearly showed that both species rich habitats and species poor habitats can support unique butterfly species depending on their resource utilization.

Habitat selection in butterflies is directly related to the availability of preferred food plants for larvae and adults (Grossmueller and Lederhouse 1987; Thomas 1995). In the present study, the maximum number of species and individuals were observed in mature mixed moist deciduous forest (TWLS I), followed by regenerated secondary mixed moist deciduous forest (TWLS II).

The degraded forest of TWLS III, with patches of woods, grasses, and herbs, supported 33 species of butterflies. The presence of plenty of woods and dense understory delimited by profuse growth of diverse herbs and shrubs in these habitats provide a rich nectar source to adult butterflies and food to larvae. The poor species richness and low diversity of butterflies recorded in the grassland habitats of TWLS IV corroborate with the findings of Ramesh et al. (2010), who recorded a similar distribution pattern of butterflies in the western coasts of India. The cultivation of exotic grass species, which creates problems for the host-specific butterfly species that rely on locally available plant species for their survival, may be responsible for the poor species richness and diversity of TWS IV. Similarly, invasive species impacted many native herbivores, particularly those species for which the plant serves as a potential food plant (Nagy et al. 1998). The high species richness, abundance, diversity, and uniqueness of TWLS I make it a key habitat for TWLS.

The results showed that more than 50% of the butterfly species recorded in all the habitat types was the same, despite differences in habitat features. The high similarity values of butterfly fauna between different habitats of TWLS indicate low beta diversity over short distances between the four transects in this forest, and bear resemblance to a study conducted in tropical forests, where herbivorous insects showed low beta diversity even over large areas (Novotny et al. 2007). It is generally accepted that rich floral diversity in tropical forests promotes herbivores, many of which are generalists (Price 1997).

Many studies have reported the relationship between habitat heterogeneity and species diversity (Bazzaz 1975; Brooks 1997; Atauri and Lucio 2001; Tews et al. 2004). In most habitats, plant communities determine the physical structure of the environment, and therefore have considerable influence on the distributions and interactions of animal species (Lawton 1983; McCoy and Bell 1991). The highest diversity, minimum dominance, and occurrence of seven unique species of butterflies recorded in TWLS I may be attributable to its vegetation complexity and multilayered canopy, which facilitate different sets of microclimates, making the habitat distinct for different butterfly species. Rosenzweig (1981) stated that diversity is enhanced by the presence of specialists that exhibit distinct habitat preferences. TWLS II also attracted 37 species of butterflies due to the edge effects of the neighboring habitats of TWLS I and TWLS III, and an additional feature of a large number of pits caused by uprooting of plants by wild boars. Pits provide sites for deposition of mineral rich feces and urine perfect for mudpuddler butterflies, such as *Papilio polytes* L., *Papilio demoleus* L., *Castalius rosimon* Fruhstofer, and *Eurema hecabe* (Moore). This finding is in agreement with the findings of Ramos (1996).

The species estimator Chao 2 order was found to be a reliable overall predictor with respect to accuracy of species richness in the four habitat types of this study. Peterson et al. (2003) and Brose et al. (2003) reported similar results using Jackknife 2 order for small sample sizes.

The findings indicated that mature secondary and regenerated forests supported high butterfly diversity and species richness, while exotic grassland had a negative impact on butterfly community. The occurrence of six threatened species and 21 unique species in TWLS strongly suggests that there is a need for the implementation of sustainable conservation strategies for the protection of the rare taxa of the sanctuary.

## Abbreviations

**TWLS**,: Trishna wildlife sanctuary
